# Thyroid Disruption in Zebrafish Larvae by Short-Term Exposure to Bisphenol AF

**DOI:** 10.3390/ijerph121013069

**Published:** 2015-10-16

**Authors:** Tianle Tang, Yang Yang, Yawen Chen, Wenhao Tang, Fuqiang Wang, Xiaoping Diao

**Affiliations:** 1 College of Environment and Plant Protection, Hainan University, Haikou 570228, China; E-Mails: ttl-0114@163.com (T.T); yangyang_0221@hotmail.com (Y.Y); yawenchen2010@hotmail.com (Y.C); twh1229@163.com (W.T); wangfuqiang1991@126.com (F.W); 2 School of Tropical and Laboratory Medicine, Hainan Medical University, Haikou 571199, China; 3 Haikou Key Laboratory of Environment Toxicology, Hainan University, Haikou 570228, China

**Keywords:** bisphenol AF (BPAF), zebrafish (*Danio rerio*), thyroid hormones (THs), hypothalamic-pituitary-thyroid (HPT) axis

## Abstract

Bisphenol AF (BPAF) is extensively used as a raw material in industry, resulting in its widespread distribution in the aqueous environment. However, the effect of BPAF on the hypothalamic-pituitary-thyroidal (HPT) axis remains unknown. For elucidating the disruptive effects of BPAF on thyroid function and expression of the representative genes along the HPT axis in zebrafish (*Danio rerio*) embryos, whole-body total 3,3′,5-triiodothyronine (TT3), total 3,5,3′,5′-tetraiodothyronine (TT4), free 3,3′,5-triiodothyronine (FT3) and free 3,5,3′,5′-tetraiodothyronine (FT4) levels were examined following 168 h post-fertilization exposure to different BPAF concentrations (0, 5, 50 and 500 μg/L). The results showed that whole-body TT3, TT4, FT3 and FT4 contents decreased significantly with the BPAF treatment, indicating an endocrine disruption of thyroid. The expression of thyroid-stimulating hormone-β and thyroglobulin genes increased after exposing to 50 μg/L BPAF in seven-day-old larvae. The expressions of thyronine deiodinases type 1, type 2 and transthyretin mRNAs were also significantly up-regulated, which were possibly associated with a deterioration of thyroid function. However, *slc5a5* gene transcription was significantly down-regulated at 50 μg/L and 500 μg/L BPAF exposure. Furthermore, *tr*α and *tr*β genes were down-regulated transcriptionally after BPAF exposure. It demonstrates that BPAF exposure triggered thyroid endocrine toxicity by altering the whole-body contents of thyroid hormones and changing the transcription of the genes involved in the HPT axis in zebrafish larvae.

## 1. Introduction

Bisphenol AF (BPAF, 1,1,1,3,3,3-hexafluoro-2,2-bis(4-hydroxyphenyl)propane), is a derivative of bisphenol A (BPA) in which the (–CH_3_) groups are replaced by (–CF_3_) groups. It is one of the high-yielding raw materials used in fluoroelastomers and polyesters [1,2,3]. Though BPAF likely has limited use in food-transfer tubing and possible use as a processing aid in polyolefins for food and drug packaging [1], knowledge about its toxicological outcome and environmental fate are still limited. BPAF has been detected at a concentration of 1.53 × 10^4^ ng/L in water and 2.00 × 10^3^ ng/g (dry weight) in sediments which were sampled from a river near a factory in China [4]. The concentrations here were higher than those found in soil (331 ng/g) and indoor dust (739 ng/g) samples. Exposure to high concentrations of BPAF may affect the environmental safety and the health of residents near the factory. The BPAF detection ratio was nearly half (24/52) in 52 samples collected from 30 urban sewage treatment plants in China, and the highest concentration detected was 45.1 ng/g (dry weight) [5]. Moreover, BPAF has also been detected in rivers, aquatic organisms, and human bodies. In urine samples, there was a concentration range from < Limit of Quantification (LOQ) to a few ng/mL in subjects near a BPAF manufacturing plant in south China [6]. BPAF has been linked to endocrine abnormalities in fish and human, resulting in increased attention to its contamination of the aquatic environment [4,5,6]. 

*In vitro* date can be part of toxicological evaluations. BPAF may pose potentially as an endocrine disruptor for animals by binding to hormone receptors [7] and by acting as a cell type-specific antagonist (≤10 nM) for ERβ in HeLa cells [8]. Previous studies have shown that BPAF can induce estrogenic actions via binding to estrogen receptor (ER) [8,9]. A recent study demonstrated that BPAF-induced endogenous transcription of estrogen responsive genes was mediated through both genomic and nongenomic pathways involving the ERα and ERK1/2 activation in human breast cancer cells [10]. Another study on human peripheral blood mononuclear cells (PBMCs) demonstrated that BPAF could cause cell viability reduction by depleting intracellular ATP levels and altering PBMC size and granulation [11]. Furthermore, BPAF is cleared slowly more than BPA in hepatocytes with the clearing rate ranking of rat > mouse > human [12]. A sharp decrease in the number of oocytes reaching maturity was also observed in mice after exposure to BPAF [13].

Thyroid hormone activity is particularly important during early development in amphibians and fishes [14]. Zebrafish (*Danio rerio*) is a vital vertebrate model organism and has been used as a model species to identify the action of endocrine disrupting chemicals (EDCs). Many studies have shown that the toxicity profiles of mammalian and zebrafish are very similar [15,16]. In zebrafish, the hypothalamic-pituitary-thyroid (HPT) axis controls thyroid endocrine system which is responsible for regulating the synthesis of thyroid hormones (THs). Previous study found thyroid hormone 3,5,3′,5′-tetraiodothyronine (T4) production starts around 72 h post-fertilization [17]. The major thyroid hormone (TH) secreted by the thyroid follicles is T4, while the majority of the most active form of TH, 3,5,3′-triiodothyronine (T3), is generated in peripheral tissues by the deiodination of T4. The thyroid endocrine system and xenobiotics in zebrafish (*Danio rerio*) have attracted increasing attention in recent years. Xenobiotics possibly act as thyroid-disruptor by disturbing TH synthesis, secretion, transport, binding, and regulation [18,19]. Thyroid stimulating hormone (TSH) secretion in teleosts is stimulated by corticotrophin-releasing factors [20]. Three types of deiodinases (*dio1*, *dio2* and *dio3*) convert T4 to T3 by removing iodine from T4 or producing metabolically inactive counterparts, thereby producing more physiologically active compounds [21]. *Dio1* is a difunctionality deiodinase as it is capable of promoting both activation and inactivation of THs. However, *Dio2* only catalyzes activation and *Dio3* merely the middle of catalyzes inactivation [22]. The main effect of *Dio3* is to deactivate T4 by inner ring deiodination (IRD), leading to the formation of the inactive 3,3′,5′-triiodothyronine (rT3) or to generate 3,3′-diiodothyronine (T2) from T3. In most fish, THs exert their effects by binding to specific TRs, such as TR-α and TR-β, which have important roles in the larval development [23]. Their structure and function closely resemble those of higher vertebrates [22].

*In vivo* assays are the most appropriate methods to elucidate the potential effects of a thyroid toxicant on the thyroid system [24]. The potential influence of BPAF on TH homeostasis in zebrafish larvae through ecotoxicological exposure to BPAF in the aquatic environment is unknown. In the present study, BPAF disruption effects on zebrafish (*Danio rerio*) reproduction were examined. The potential toxicity effects of BPAF on the hormonal balance and genes of the hypothalamic HPT axis of zebrafish larvae were determined. The enzyme-linked immunosorbent assay (ELISA) was employed to measure the concentrations of the physiologically relevant total 3,3'5-triiodothyronine (TT3), total 3,5,3′,5′-tetraiodothyronine (TT4), free 3,3'5-triiodothyronine (FT3) and free 3,5,3′,5′-tetraiodothyronine (FT4) after BPAF exposure to verify the influence of BPAF on TH homeostasis in zebrafish larvae. Finally, the expression of relevant genes involved in the HPT axis was also analyzed with particular emphasis on the pathways of TH synthesis.

## 2. Experimental Section 

### 2.1. Reagents and Preparation of Stock Solutions

BPAF (CAS no.1478-61-1; ≥99.5% purity) was purchased from Xiya Reagent (Shandong, China), while dimethyl sulphoxide (DMSO; CAS no.67-68-5; ≥99.5% purity) was from Solarbio (Beijing, China), sea salt from Instant Ocean (Ohio, OH, USA), methane-sulfonate salt (MS-222) from Sigma (St. Louis, MO, USA). BPAF stock solutions (50 mg/L, 5 mg/L and 0.5 mg/L) were prepared by dissolving BPAF in dimethyl sulphoxide (DMSO) (100 mL), followed by dilution to 1 L with pure water containing 60 mg Instant Ocean sea salt, respectively. These solutions were further diluted to 5, 50 and 500 µg/L and named as the second stock solutions. Test water was prepared by dissolving 60 mg of Instant Ocean sea salt in 1 L of pure water to prevent interference by impurities in tap water. DMSO solvent (0.1%, treated as control) was freshly prepared by adding 1 mL of DMSO in 1 L of test water [25]. BPAF stock concentrations were confirmed by high-performance liquid chromatography (HPLC) analysis. Because BPAF has a half-life of about 180 days in water [26], it has no problem to carry out the experiment for 168 h.

### 2.2. Fish Maintenance

The test was initiated with fertilized eggs of zebrafish (*D. rerio*) obtained from non-exposed adults (AB strain, aged 3 months). Natural crosses were adopted. Throughout the study, adult fish were maintained under an artificial light/dark period of 14/10 h and a constant temperature (28 ± 1 °C) in an aerated aquarium system. Adult fish were fed twice daily with a commercial flake food (Charoen Pokphand Group, Bangkok, Thailand) complemented with freshly hatched *Artemia nauplii* (Charoen Pokphand Group) without any solvent or BPAF. The fertilized eggs were examined with a dissecting microscope. Those eggs reached the blastula stage (2 h post-fertilization) were selected for subsequent experiments.

### 2.3. Experimental Design

The experiment was designed and improved according to Organization for Economic Co-operation and Development (OECD) Test Guideline 236 with semi-static test. Beakers (500 mL) were filled with freshly prepared test solutions (300 mL), which were renewed by adding freshly prepared test solutions (150 mL) every 12 h to keep the BPAF concentration constant [27]. The treatments included 5, 50 and 500 µg/L BPAF exposure and the solvent control (SC). The selected concentrations were ascertained by our previous study. The highest concentration was based on 25% of the concentration for 50% of maximal effect (EC_50_) (based on the malformation rate, 24 h EC_50_ of BPAF was 2.00 mg/L for the embryos) [28]. The lowest concentration was based on 1/3 of an environmental investigation concentration [2]. Each treatment group was replicated thrice and held a starting number of 300 randomly selected fertilized eggs per replicate (therefore have 900 individuals per treatment). All groups were incubated under constant temperature (28.5 °C) and humidity (70%) with 14/10 h light/dark period. The beakers were wrapped with a food wrapper before placing in the incubator. During the experimental period, embryos and hatched larvae were not fed, and the shed chorion and dead larvae were immediately removed. The hatch time and survival rates of embryos were recorded. After 168 hpf, zebrafish larvae were anesthetized with MS-222 (0.03%) and sampled for body weight length determination, thyroid hormones measurement, and gene expression analysis. 

Animal welfare and experimental procedures were carried out in accordance with the Guide for the Care and Use of Laboratory Animals (China National standardizing committee GB 14925-2010 and Ministry of Science and Technology of China, 2006), and were approved by the animal ethics committee of Hainan Medical University.

### 2.4. Hormone Measurements

A total of 200 larvae from each replicate were sonicated to 5% (*w*/*v*, g/mL; *w*: wet weight of 200 larvae, *v*: volume of 0.01 M PBS, pH 7.2) whole-body homogenate at 0 °C. The homogenate was centrifuged at 12,000 × *g* for 5 min at 4 °C. The supernatant was collected for measuring the whole-body levels of TT3, FT3, TT4, and FT4 using enzyme-linked immunosorbent assays (ELISA, commercial kit for fish), respectively. The commercial kits for TT3, TT4, FT3, and FT4 were purchased from X-Y Biotechnology (Shanghai, China) and manufacturer’s instructions were followed. The assay detection limits were 0.4 ng/mL for TT3, 3 ng/mL for TT4, 0.13 pg/mL for FT3 and 0.08 pg/mL for FT4, respectively. Intra-assay and inter-assay variations were below 15% in this study. No significant cross-reactivity or interference was observed for each kit.

### 2.5. mRNA Expression of Selected Genes

Residual larvae from each replicate were homogenized with disposable tissue grinding pestles. The homogenate was prepared for RNA extraction using Trizol reagent (Invitrogen, Carlsbad, CA, USA). Total RNA concentration was estimated based on the results of NanoDrop 2000 (Thermo, Logan, UT, USA). The RNA quality was examined by measuring the 260/280 nm ratios (1.98–2.05) and 0.8% agarose-formaldehyde gel electrophoresis with GoldView™ staining. The synthesis of first-strand complementary DNA (cDNA) was performed by using M-MLV reverse transcriptase (Promega, Madison, WI, USA) with 2 µg RNA reverse-transcribed in each sample. Quantitative real-time polymerase chain reaction (qRT-PCR) was performed using a SYBR^®^ Green PCR kit (Applied Biosystems Inc., Carlsbad, CA, USA). The primers were obtained from Sangon Biotech (Shanghai, China). The forward and reverse primer sequences for genes (β*-actin*, *tsh-*β, *dio1,* *dio2*, *sclc5a5*, *tg*, *ttr*, *tr-*α, and *tr-*β) are shown in Table 1. Moreover, 1.5% agarose gel electrophoresis of the PCR products was performed to confirm primer quality and the presence of single amplicons of the correct predicted size (100bp). qRT-PCR was performed using a LightCycler96 PCR instrument (ROCHE, Basel, Switzerland) with a 2× SYBR^®^ Green qPCR Mix kit (Applied Biosystems Inc., Carlsbad, CA, USA). The thermal cycle was set at 95 °C for 10 min, followed by 40 cycles of 95 °C for 45 s, 60 °C for 30 s, and 72 °C for 30 s. Before our mRNA expression experiment, we assessed the amplification efficiencies of primers and transcriptional stability of three candidate genes (*rpl8*, *18s*, β*-actin*) commonly used as reference genes for BPAF from exposure to a single compound. The results of the analysis showed that the β*-actin* was the most stable gene for BPAF single treatment and β*-actin* was selected as the reference gene for the mRNA expression assay in this study. The mRNA expression of each target gene was normalized to β*-actin*. β*-Actin* transcript was used to standardize the results by eliminating variations in mRNA and cDNA quantity, as it was did not vary upon chemical exposure (data not shown) and was used as internal control. To ensure that a single product was amplified, melting curve was used to examine the specificity of PCR products. After PCR, the melting curve was a single peak, demonstrating the specificity of the PCR product. The transcription levels of target genes (*tsh-*β*,* *dio1, dio2, sclc5a5, tg, ttr,* *tr-*α*,* and *tr-*β) were determined in duplicates. The relative expression levels were calculated using the 2^−^^△△^^Ct^ method [29].

### 2.6. Statistical Analysis of Data

The data of the developmental parameters and hormone assay are presented as the mean ± standard error of the mean (SEM). The differences between the control and each exposure group were evaluated by Kruskal-Wallis test (nonparametric test) followed by Nemenyi multiple comparison test. These statistical tests were conducted using SPSS for Windows 13.0 Software (SPSS, Chicago, IL, USA). Homogeneity of variances and normality of the data were analyzed by Levene’s test and Kolmogorov-Smirnov test. Statistical analyses were performed by using GraphPad Prism 6.01 computer program (GraphPad Software, Inc., La Jolla, CA, USA) and the rest of the data were also presented as mean ± standard error of the mean (SEM). The differences in each observation were evaluated by one-way ANOVA followed by Dunett’s test for identifying the differences between exposure groups and the control group. The differences were considered significant at two different levels (* *p* < 0.05; ** *p* < 0.01) relative to the controls.

**Table 1:** Primers used for the quantification of mRNA expression by qRT-PCR.

Name	Sequence of Forward (FP) and Reverse Primers (RP)	Genbank Accession
β*-actin*	FP 5′-CGAGCAGGAGATGGGAACC-3′ RP 5′-CAACGGAAACGCTCATTGC-3′	AF057040
*tsh-*β	FP 5′-GCAGATCCTCACTTCACCTACC-3′ RP 5′-GCACAGGTTTGGAGCATCTCA-3′	AY135147
*dio1*	FP 5′ -GTTCAAACAGCTTGTCAAGGACT-3′ RP 5′ - AGCAAGCCTCTCCTCCAAGTT-3′	BC076008
*dio2*	FP 5′-GCATAGGCAGTCGCTCATTT-3′ RP 5′-TGTGGTCTCTCATCCAACCA-3′	NM212789
*sclc5a5*	FP 5′-GGTGGCATGAAGGCTGTAAT-3′ RP 5′-GATACGGCATCCATTGTTGG-3′	NM001089391
*tg*	FP 5′-CCAGCCGAAAGGATAGAGTTG-3′ RP 5′-ATGCTGCCGTGGAATAGGA-3′	XM001335283
*ttr*	FP 5′-CGGGTGGAGTTTGACACTTT-3′ RP 5′-GCTCAGAAGGAGAGCCAGTA-3′	BC081488
*tr-*α	FP 5′-CTATGAACAGCACATCCGACAAG-3′ RP 5′-CACACCACACACGGCTCATC-3′	NM131396
*tr-*β	FP 5′-TGGGAGATGATACGGGTTGT-3′ RP 5′-ATAGGTGCCGATCCAATGTC-3′	NM131340

## 3. Results 

### 3.1. Developmental Parameters of Zebrafish Larvae

No significant effects were observed with survival, length, weight after exposure to BPAF (SC, 5, 50, and 500 µg/L) from being hatched to 168 hpf. However, in zebrafish embryos, hatchability of 72 hpf was significantly increased in 50 and 500 μg/L exposure groups compared with the SC (Table 2).

**Table 2:** Survival, body length, weight, and hatchability of zebrafish larvae after exposure to BPAF (SC, 5, 50, and 500 µg/L) from 72 to 168 hpf.

BPAF (µg/L)	SC	5	50	500
168 hpf Survival (%)	86.2 ± 2.1	83.5 ± 1.8	87.2 ± 0.8	85.7 ± 2.8
168 hpf Length (mm)	3.62 ± 0.08	3.60 ± 0.10	3.55 ± 0.05	3.58 ± 0.06
168 hpf Weight (mg)	0.41 ± 0.02	0.40 ± 0.03	0.38 ± 0.02	0.37 ± 0.04
72 hpf Hatchability (%)	80.5 ± 2.0	78.2 ± 1.5	44.3 ± 1.8 *	40.7 ± 1.6 *
96 hpf Hatchability (%)	92.4 ± 1.2	91.5 ± 1.3	90.8 ± 0.8	93.2 ± 1.1

The values represent as mean ± standard error (SEM) of three replicate groups. Asterisk indicates significant difference compared with solvent control (SC) (* *p* < 0.05).

### 3.2. Concentration of Hormones in Zebrafish Larvae

**Figure 1:**
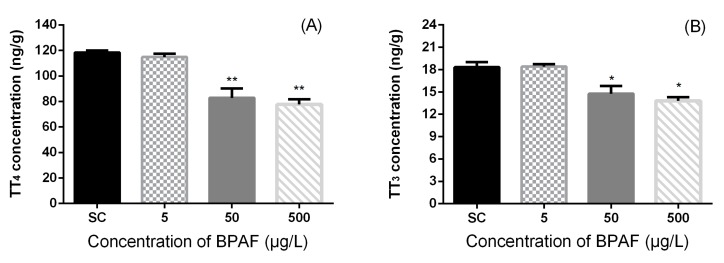
Total T4 (**A**) and total T3 (**B**) levels in zebrafish exposed to SC, 5, 50 and 500 μg/L of BPAF solution at 168 hpf. The results are shown as mean ± standard error of mean (SEM) (fifty fish as a pool, *n* = 3). Asterisks indicate statistically significant differences from solvent control (SC: DMSO 0.1%) (* 0.01 < *p* < 0.05, ** *p* < 0.01).

BPAF exposure caused significant alterations in zebrafish whole-body T4 and T3 contents at 168 hpf (Figure 1). The concentrations of TT4 and TT3 were significantly affected by different treatment (Kruskal-Wallis test, *p* = 0.0032 and *p* = 0.0108, respectively). The TT4 contents significantly decreased by 30.08% and 34.44% at 50 and 500 μg/L exposure groups, respectively, in comparison with the SC group. The concentrations of whole-body TT3 in tissue homogenate were significantly reduced by 19.42% and 24.71% at 50 and 500 μg/L exposure groups, respectively, in comparison with the SC group. However, no significant differences were observed at the 5 μg/L exposure group.

**Figure 2:**
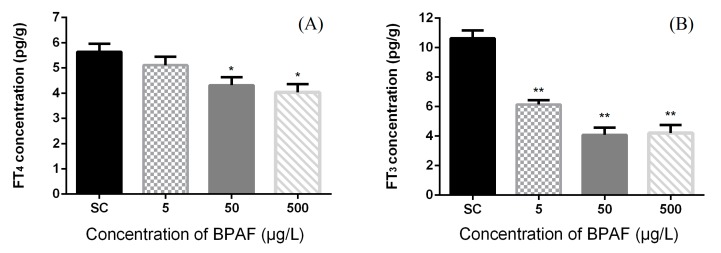
Effects of BPAF on (**A**) free T4 (FT4) hormone concentration and (**B**) Free T3 (FT3) hormone concentration in zebrafish at 168 hpf. The results are presented as mean ± standard error of mean (SEM) (fifty fish as a pool, *n* = 3). Asterisks indicate statistically significant differences from solvent control (SC: DMSO 0.1%) (* 0.01 < *p* < 0.05, ** *p* < 0.01).

The concentrations of FT4 and FT3 were significantly affected by different treatment (Kruskal-Wallis test, *p* = 0.0205 and *p* = 0.002, respectively). Exposure to high concentrations (50 and 500 μg/L BPAF) significantly reduced whole-body FT4 contents (by 22.46% and 28.55%, respectively) (Figure 2).

In comparison with SC, the FT4 levels were unchanged at 5 μg/L BPAF exposure group. Nevertheless, a reduction of FT3 levels was observed at all exposure groups, which was extremely significant in every exposure group. The basal FT3 contents in the control zebrafish was 10.637 ± 0.527 pg/g, and were significantly reduced at different treatments: 42.23% reduction, 6.145 ± 0.286 pg/g at 5 μg/L group; 61.56% reduction, 4.089 ± 0.490 pg/g at 50 μg/L group; 60.53% reduction, 4.198 ± 0.556 pg/g at 500 μg/L group (** *p* < 0.01).

### 3.3. Effects of BPAF on HPT Gene Expression Levels in Zebrafish Larvae

**Figure 3:**
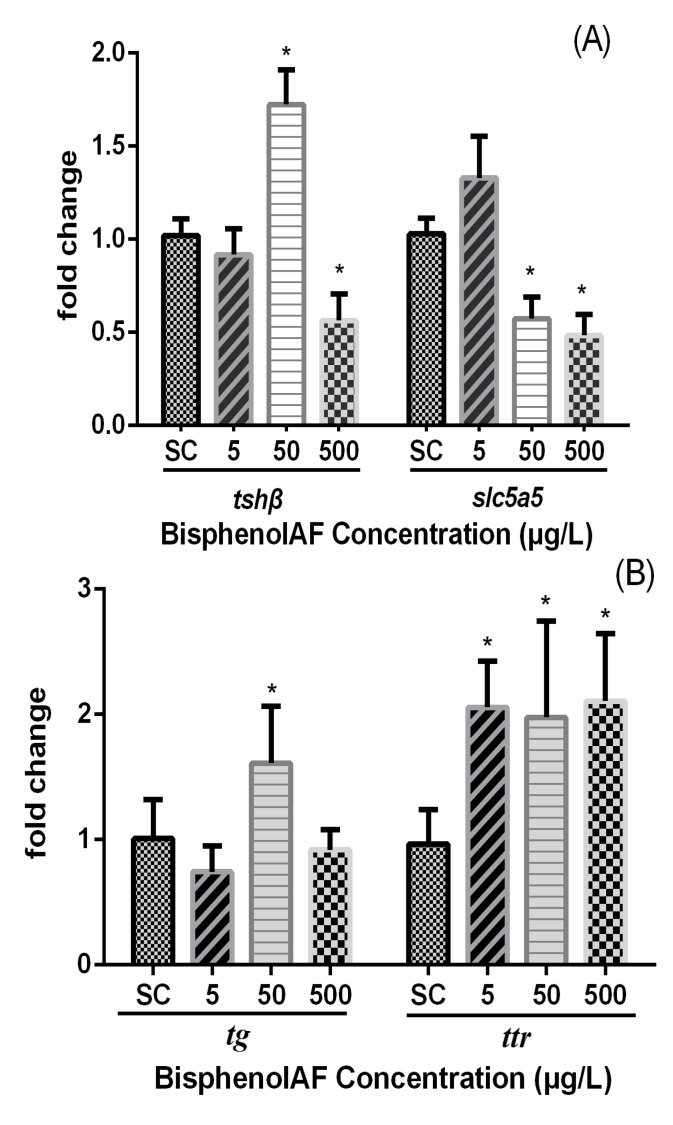

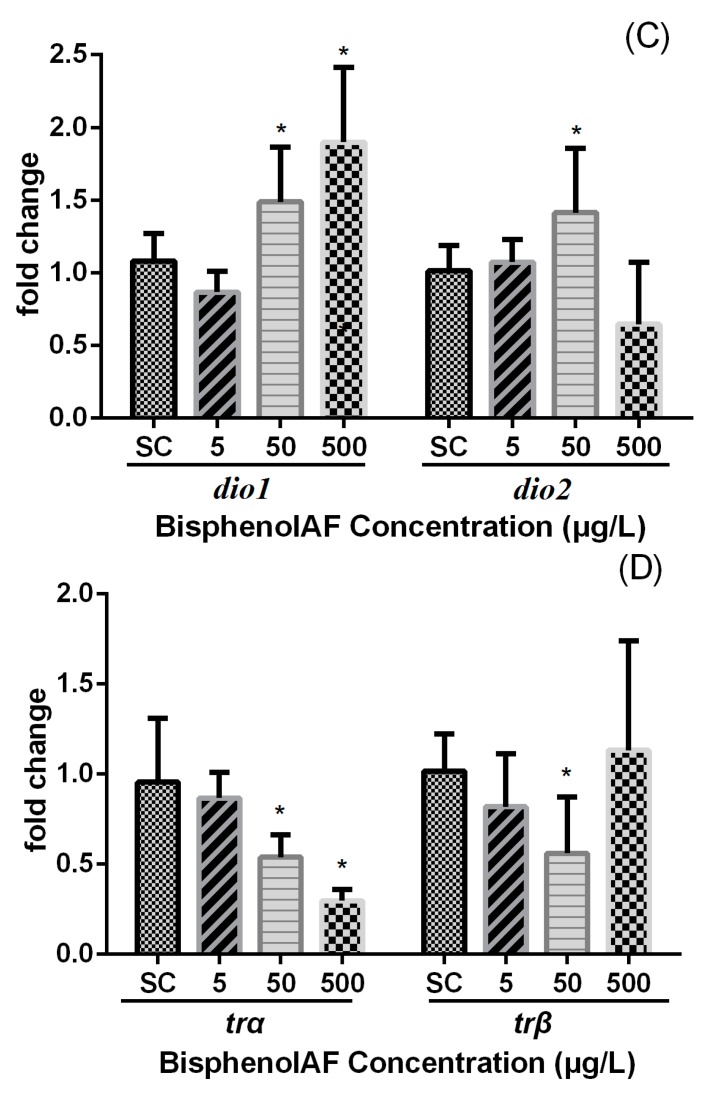
Relative mRNA expression levels of (**A**) *tsh*β and *slc5a5,* (**B**) *tg* and *ttr,* (**C**) *dio1* and *dio2,* (**D**) *tr*α and *tr*β in zebrafish larvae exposed to different concentrations of BPAF at 168 hpf (SC: 0.1% DMSO, 5, 50 and 500 μg/L, respectively). Fold change (Y axis) represents the expression of the target gene mRNA relative to that of the whole-body control group (equals 1 by definition). Data expressed as the means ± standard error of mean (SEM) (*n* = 6). Asterisks (*) represents *p* < 0.05 for significant difference.

The modification of hypothalamic-pituitary-thyroid axis (HPT) gene mRNA levels in zebrafish larvae exposed to BPAF is shown in Figure 3. The transcription of *tsh-*β gene was significantly up-regulated by 72.4% upon exposure to 50 μg/L of BPAF (0.01 < *p* < 0.05) whereas a highly significant decrease was observed at 500 μg/L. The *slc5a5* gene transcription was significantly down-regulated 44.3% and 56.1% at 50 and 500 μg/L BPAF exposure groups, respectively (Figure 3A).

The mRNA expression of gene encoding thyroglobulin (*tg*) was up-regulated transcriptionally by 68.8% whereas the transthyretin (*ttr*) gene was significantly induced by 112.8%, 104.5%, and 118.1% at a concentration-dependent manner after exposure to 5, 50, and 500 μg/L BPAF (Figure 3B). 

The expression of *dio1* significantly increased at 50 and 500 μg/L BPAF exposure in comparison with SC (*p* < 0.05). In addition, the transcription of *dio2* gene was significantly up-regulated by 40.4% after exposure to 50 μg/L BPAF (Figure 3C).

Two thyroid hormone receptors (*tr-*α and *tr-*β) were examined. The transcription of *tr-*α gene significantly decreased by 43.6% and 64.4% at 50 and 500 μg/L BPAF groups, respectively (Figure 3D), whereas significant down-regulation of *tr-*β mRNA levels were observed at 50 μg/L in comparison with the control.

## 4. Discussion

In the current study, BPAF was examined in terms of its disruption of the endocrine system, particularly the expression of selected genes along the endocrine system. Previous studies showed that other bisphenol analogues, such as bisphenol F, tetrabromobisphenol A (TBBPA), and bisphenol B, may have similar endocrine-disrupting effects based on their structural similarity with BPA. Exposure to BPAF triggers reproductive toxicity which negatively influence zebrafish offspring. However, disrupting effects of BPAF on the HPT axis are still unknown [1,2]. 

A recent study showed that the hatching delay was observed in a 5 μg/L BPAF exposed group [2]. Similar results were observed at higher concentrations of exposure in the present study. Interestingly, BPAF just delay the incubation period, but does not affect the survival rate. Therefore, BPAF may have a potential impact on embryonic activity.

Thyroid hormones play important roles in the development, growth and metabolism in teleosts. The HPT axis of fish operates by secreting hormone from the hypothalamus, and in turn stimulates the secretion of TSH that regulates the synthesis of THs, in particular T4. Thyroid function in *Anguilla japonica* was observed to be active both during and after metamorphosis [30]. Exposure to decabromodiphenyl ether resulted in alterations of both T3 and T4 levels in zebrafish [31]. A decrease in plasma T3 levels is mostly caused by a reduction of thyroidal T4 production [32]. T3 is known to be more physiologically active than T4 [21].

Some xenobiotics such as mono(2-ethylhexyl) phthalate can decrease T4 contents and increase T3 contents [33]. Inconsistent results from previous studies are likely due to the differences in xenobiotics and the concentrations used. In our previous study, the heart rates of zebrafish embryos were significantly reduced after 72 h of BPAF exposure [28]. This heart rate reduction may be related to the secretion of TH. Nevertheless, it does not fully explain the thyroid-disrupting properties of BPAF [34].

Zebrafish (*D. rerio*) embryos were exposed to different concentrations of BPAF from 2 to 168 hpf. The results showed that BPAF exposure induced a reduction in whole-body TT4 and TT3 levels, as well as whole-body FT4 and FT3 contents. The ratio of (TT3/TT4) showed no significant difference after BPAF treatment, indicating the relative normal of TH homeostasis in the larvae after exposure to BPAF. However, the concentrations of FT3 and FT4 were significantly reduced in exposure groups compared to the SC. It might result in a reduction in the amount of THs available for the target tissues, by which some vitally metabolic pathways such as energy metabolism might be compromised [35]. T3 is generated in peripheral tissues by deiodination of T4. Therefore, a reduced T3 levels is mostly due to a drop in thyroidal T4 production and secretion and/or the changes of THs metabolism [32]. Iodothyronine deiodinases play important roles in the mechanism of thyroid hormone biotransformation.

Three types of *dio* genes can be found in teleosts: *dio1, dio2,* and *dio3*. Each of these genes plays an important role in the regulation of fish circulation [36]. In particular, *dio2* is responsible for activating the outer ring-deiodination pathway by converting T4 to T3 [21]. Hypothyroidism has been recently demonstrated to increase *dio2* mRNA expression [21]. An increase of *dio2* mRNA level following the developmental exposure to 50 μg/L BPAF was observed, which is consistent with a previous study [37]. *Dio3* merely the middle of catalyzes inactivation is not involved in the present study. *Dio1* plays insignificant role in TH homeostasis, but it has a big influence on iodine recovery and TH removal. In addition, up-regulated *dio1* mRNA levels were observed at 50 and 500 μg/L BPAF. Previous studies suggest that modification in the transcription of *dio2* might be responsible for the decrease of T4 levels. Increased transcription of *dio1* might assist to reduce the raised T3 contents as a compensatory mechanism in larvae [33]. These results showed that BPAF could cause hypothyroidism in zebrafish larvae.

TSH is a glycoprotein that consists of α-subunits and β-subunits, and could be used to analyze the integrity of pituitary and thyroid function [24]. Furthermore, the β-subunit significantly determines the hormone’s functional specificity [38]. *Tsh-*β mRNA levels have been observed to be up-regulated in goldfish after exposure to the pesticide monocrotophos [39]. Exposure to 10 µg/L of DE-71 significantly increased the transcription of *tsh-*β genes [40]. Increased *Tsh-*β mRNA levels have been observed in the embryo up to the larval stages in zebrafish after exposure to BPA. Conversely, a highly significant decrease has been observed after exposure to TBBPA [41]. In the present study, the changes in the mRNA expression of the *tsh-*β gene demonstrated that *tsh-*β mRNA levels in larvae increased after exposure to 50 μg/L of BPAF, but down-regulated in a 500 μg/L BPAF exposure group. The pituitary gland regulates thyroid activity through the secretion of TSH, and uses a negative feedback mechanism to reduce T4 [42]. The present results showed that the increase of the *tsh-*β mRNA contents might be attributed to the reduced negative feedback from the hypothalamus. TSH synthesis and release are controlled by two major factors: T3 level and thyrotropin releasing hormone (TRH). TSH synthesis and release can be stimulated by low levels of THs in hypothyroidism, which could explain the up regulation of TSH and inhibition of T3 level at 50 μg/L treated embryo/larvae. Such pituitary activity was due to decreased level of circulating T4 [40]. However, the decrease of TSH-β mRNA levels caused by 500 μg/L BPAF exposure may be due to the injury of pituitary gland or the deletion of the follicle colloid lumen in zebrafish (*D. rerio*) larvae. 

Known to encode a sodium/iodide symporter, the slc5a5 gene is expressed in the thyroid and plays an important role in taking iodine from the bloodstream in zebrafish [43]. The slc5a5 gene was involved in TH synthesis pathways [44]. The results of the present study suggest that the transcription of slc5a5 gene was significantly down-regulated. It is consistent with previous studies on zebrafish [41], and may be a result of structural similarity. The reduction in slc5a5 gene expression will reduce iodide transport. As a result, the thyroid gland cannot accumulate iodide, which decreases T4 production.

Exposure to 50 µg/L concentrations of BPAF can up-regulate thyroglobulin (TG) transcription. Similar results have been reported in fish larvae exposed to other xenobiotics [41,45,46]. TG is a scaffold protein which can be used as a biomarker for the detection of thyroid activity [47]. The changes of *tg* mRNA levels may act as a trigger for variations in thyroid stimulation and thyroid damage. The fact of slightly increased *tg* mRNA at 50 μg/L is perhaps consistent with reduced slc5a5 gene expression. If iodide uptake was compromised and T4 synthesis reduced one might expect to see even if transiently or just at one dose an accumulation of TG.

TTR is a transport protein and is mainly synthesized in the liver of fish. It can non-covalently bind with most THs in the blood and regulate the supply of the hormone to vital tissues [48,49]. Previous studies showed that some xenobiotics are highly competitive for free TH-binding to TTR *in vitro* [50]. In a recent study, down-regulated TTR gene expression in association with exposure to some pollutants reduced the amount of TTR binding to free THs, resulting in fewer TTR protein translations, which partly inhibited the sharp decline in free TH levels [39]. In contrast, the present study shows that the decreased whole-body TT3 levels and up-regulated *ttr* gene expression in exposed groups may lead to a decrease of FT3 levels. Furthermore, lower FT3 content would have less influence to energy metabolism. 

TRs, including TR-α and TR-β isoforms, act as inducible ligand-activated transcription factors and play important roles in embryonic and larval development [23]. Recent work reported that adult female fathead minnows show decreased levels of T4 and down-regulated *tr-*β gene expression when exposed to BDE-47 [51]. In the present study, down-regulation of *tr-*α and *tr-*β gene expression were observed, illustrating the disrupting effect of BPAF on the TR signaling system during larval development.

## 5. Conclusions

The study indicated a no-effect BPAF level of 5 μg/L. Exposure of fertilized embryos to high concentrations of BPAF would result in delayed hatching and impact the development of offspring. The exposure of zebrafish to BPAF larvae resulted in decreased TT4, TT3, FT4 and FT3 levels by altering the expression of relevant genes involved in the HPT axis. Free hormone including FT3 and FT4 levels are helpful biomarkers in teleosts, due to the potentially physiologically function in organism. The mRNA expression of the genes that regulate TH synthesis and transport at multiple sites of the HPT axis were used as potential biomarkers. However, the effective concentrations of BPAF were much higher than that in common environment. Thus, short-term exposure to BPAF alone at a common environmental concentration (15 µg/L) may not cause thyroid endocrine disruption in teleosts. BPAF could cause changes not only limited to the HPG axis but also certain disruptions of the HPT axis. This information would be helpful in providing a further theoretical basis for ecological risk assessment. At present, there were few reports about BPAF in the environment, so the lakes, rivers and other locations to establish the range of values in the environment deserve further investigation in the future.
